# Altered surface behaviour in earthworms (*Lumbricus terrestris*) under artificial light at night

**DOI:** 10.1007/s00442-025-05750-z

**Published:** 2025-06-27

**Authors:** Jiaqing Cai, Jonathan Bennie, Kevin J. Gaston

**Affiliations:** 1 Environment and Sustainability Institute, University of Exeter, Cornwall, Penryn, TR10 9FE UK; 2 Centre for Geography and Environmental Science, University of Exeter, Cornwall, Penryn, TR10 9FE UK

**Keywords:** Artificial light at night, Behaviour, Carbon cycling, Earthworms, Ecosystem processes, Light pollution

## Abstract

Artificial light at night (ALAN) has been spreading rapidly globally, disrupting animal physiology, behaviour and associated ecosystem processes. However, impacts on soil-dwelling animals that are nocturnally active aboveground remain underexplored. Here, we examined these for the earthworm *Lumbricus terrestris* L., an ecosystem engineer that frequently surfaces at night. We recorded four types of *L. terrestris* nighttime surface behaviour, including overall surface activity, and three finer categories: risk exploration before surfacing, foraging and over-surface exploration. One experiment (controlled-temperature-room-based) tested these behavioural responses to seven ALAN levels, whilst another (field-based) investigated whether behavioural responses can consequently affect litter removal and soil respiration. In the controlled-temperature room, compared to darkness, *L. terrestris* significantly decreased their overall nighttime foraging at ALAN levels of 10 lx and above, with these negative ALAN effect sizes increasing with light intensity. With more risk exploration before surfacing, *L. terrestris* still showed greatly reduced foraging activity at the 10 lx ALAN level in the field. However, such altered behaviour did not cause clear patterns in either litter removal or soil respiration. Our results confirm that 10 lx ALAN (or potentially lower) is effective in disrupting *L. terrestris* nighttime surface behaviour, but this may have limited impacts on short-term C cycling.

**Supplementary Information:**

The online version contains supplementary material available at 10.1007/s00442-025-05750-z.

## Introduction

The use of artificial sources has widely introduced light into nighttime land and seascapes in places, times and forms that it has not previously occurred (Gaston et al. 2021); skies have been brightened above *c.* 23% of the world’s land surface (Falchi et al. 2016; Kyba et al. 2023). This has undoubtedly brought enormous benefits to humankind; however, artificial light at night (ALAN) has also had impacts on many other organisms. Such impacts have now been documented at the levels of individuals (e.g. physiology, behaviour), populations (e.g. abundance, distribution, vital rates), communities (e.g. species richness and composition) and ecosystems (e.g. functions and services; Gaston et al. 2013; Sanders et al. 2021). They have been documented across microbes, fungi, plants and animals (Gaston et al. 2013; Hölker et al. 2015; Bennie et al. 2016), and in terrestrial, freshwater and marine environments (Sanders et al. 2021; Gaston and Sánchez de Miguel 2022). Indeed, the number of studies documenting effects of ALAN, and the range of organisms and circumstances in which these have been shown to occur, continue to grow rapidly.

This said, important gaps in understanding persist. One of these concerns the potential impacts of ALAN on soil organisms, and the ecosystem functions that they perform, which have received little attention. On the one hand, this is perhaps not a surprise, as soil is an efficient light barrier (light penetration is up to 4–5 mm into soil; Tester and Morris 1987), and thus direct impacts of light on belowground organisms might be expected in general to be minor. However, exceptions might be those soil organisms that are active on the soil surface during the night, and this might be of concern where the above or belowground activities they perform have significant ecological effects.

One case that may be of particular interest is the earthworm *Lumbricus terrestris*. This is a deep-burrowing species that has long been known to be highly light-sensitive dependent on illumination level and/or spectrum (Hess 1924; Walton 1927), and that regularly visits the soil surface for foraging, mating activity and/or dispersal (Darwin 1892; Nuutinen and Butt 1997). A substantial body of studies has found adverse effects of artificial light on *L. terrestris* behavior (e.g. withdrawal—Peeke et al. 1965; crawling—Kirk and Thompson 1967; burrowing—Gardner and Ratner 1970). This work was almost invariably motivated by interest in behavioural responses and not by interest in ALAN (only two studies, to our knowledge, were ALAN-related: Kavassilas et al. 2024; Mittmannsgruber et al. 2024), and studies predominantly used intensely bright lights to mimic daylight conditions (Wayner and Zellner 1958), or lights that also produced heat (e.g. incandescent lamps; Marian and Abramson 1982), thereby potentially confounding light and temperature effects. A more recent study by Nuutinen et al. (2014) has also revealed that, though under naturally high levels of illumination, *L. terrestris* surface behaviour was strongly limited by nighttime lighting.

As an ecosystem engineer with anecic strategies, *L. terrestris* can cause profound changes in the associated ecosystem processes (Edwards and Bohlen 1996). It has been suggested that *L. terrestris* (unit: g^−1^ fresh weight) can remove leaf litter at rates of 2.6 to 16.5 mg dry weight day^−1^ (Raw 1962; Curry and Bolger 1984), notably stimulating nutrient turnover at an estimated organic C sequestration rate of 22 g C m^−2^ yr^−1^ (Shuster et al. 2001; Don et al. 2008). Whilst this process contributes to soil C stock, it is likely accompanied by more CO_2_ output (~ 13–32% higher on average; Nieminen et al. 2015). This is of particular concern as *L. terrestris* is thought to be instrumental in C stabilisation (Sheehy et al. 2019), juxtaposed with some claims that C mobilisation prevails (Marhan and Scheu 2005a). Previous research has linked this to contextual differences (e.g. climate and soil properties; Versteegh et al. 2014) and the complex interactions with soil micro-organisms (Hoang et al. 2020) or anthropogenic drivers, such as tillage (Nieminen et al. 2015). At this point, however, it remains unclear whether there exists a path for another widespread anthropogenic driver (i.e. ALAN), potentially to influence litter removal and CO_2_ emissions through altering *L. terrestris* surface behaviour.

Here, we report the results from two experiments conducted using *L. terrestris* to determine (i) whether ALAN at levels that occur in real-world artificial nighttime lighting scenarios reduce earthworm activity at the soil surface; and (ii) whether such reduction further translates into decreased litter removal and soil respiration.

## Materials and methods

### Materials

Healthy adult *Lumbricus terrestris* were employed as the focal organisms, sourced from a domestic supplier (Wiggly Wigglers, Herefordshire, UK).

Leaf litter comprising a mix of *Quercus robur* and *Tilia cordata* was selected as a food source, due to its comparably high C:N ratios and rich Calcium, indicators that are positively correlated with *L. terrestris* abundance (Hobbie et al. 2006). Brightly coloured, freshly fallen leaf litter with plump petioles and no signs of decomposition was collected on Penryn Campus, University of Exeter (50°10' N, 5°7' W). The litter was transported back to the lab, sorted by species and dried in a forced-air oven at 40 °C for 1 week.

Field-collected topsoil (0–20 cm) was obtained from the research site on the University of Exeter’s Penryn Campus (50°10' N, 5°8' W). This soil was classified as sandy loam with 52% sand, 46% silt, 2% clay and a pH-CaCl_2_ of 4.8. Collection occurred in areas indicative of an earthworm-friendly dwelling environment. Soils were air-dried, finely sieved (2 mm) and repeatedly mixed to create a homogenous substrate.

### Mesocosms

As experimental units, mesocosms were constructed using polyvinyl chloride pipes (50 cm in height, 15.5 cm in diameter) and cleaned with 70% ethanol. The bottom of each pipe was fitted with a perforated plastic cap where 1 cm high 2–4 mm granules of sand were filled, and a metal mesh sheet with 1 × 1 mm apertures was placed, to allow drainage with minimal soil loss. All pipes were filled with air-dried soil to a depth of 40 cm and static-cultured for 1 week, during which time each was topped up with additional soil by equal mass, to ensure a uniform depth throughout the experiment (air-dry bulk density of 0.93 g cm^−3^). Inside the top of each pipe, a strip of daylight white (correlated colour temperature = 6086 K, Fig. S1) light-emitting diodes (LEDs) were secured to the wall, 5 cm above the soil surface. The LED strip light was powered by the mains and controlled by a light-sensitive switch to turn on at 70 lx and off at 110 lx. Each pipe was placed into an empty 33 × 27 cm (inner diameter × height) bucket, to capture (if any) escaped worms. Prior to initiation of the experiments, all mesocosms were saturated with water, and soil water content was adjusted to 60% of the maximum water-holding capacity.

### Experimental design

#### Experiment 1

Experiment 1 was a repeated measures design where four mesocosms were set up and subjected to seven ALAN treatments (i.e. 0.1, 1, 10, 20, 50, 100 lx and a dark control) in a controlled-temperature room. Low level light treatments (0.1 and 1 lx) simulated city skyglow levels and levels away from the immediate vicinity of streetlights, medium light level treatments (10 and 20 lx) simulated levels in close proximity to streetlights, and high light level treatments (50 and 100 lx) simulated more extreme lighting comparable to stadium or festival illumination. To avoid potential light adaptation in earthworms, each night one of the seven ALAN treatments was randomly chosen and applied to all mesocosms (Fig. S2). Nights for each ALAN treatment were replicated five times. Illuminance was adjusted using a standard hand-held lux metre (DT-8820 Environment Meter, CEM, Shenzhen, China; illuminance sensitivity, 0.01–20000 lx; accuracy, ± (5.0% of reading + 10 digits)).

Before initiation of the experiment, earthworms were pre-treated for a week: kept in groups of 10–20 individuals in 10 L buckets with regularly re-moistened soils, fed leaf litter ad libitum and cultured under 15:9 h day-night cycles. One day prior to introduction to mesocosms, earthworms were kept in wet paper towels for 24 h to allow gut voiding. After the starvation procedure, earthworms were weighed (average live mass 3.84 g each) and distributed evenly by weight to the mesocosms to control for any size effects. Each mesocosm was inoculated with six earthworms, which falls within the field population density as estimated by Kammenga et al. (2003). After inoculation for a week to allow habituation with the repacked soil environment, the mixed leaf litter was introduced (litterbag-free, 0.1 g every 3 days to the surface centre).

To record earthworm nighttime behaviour on the soil surface, a webcam was used (TapoTC65, TP-Link USA Corp., Irvine, CA). This was secured above the pipe and pointed downwards to offer a general view of the soil surface.

This experiment was carried out in a day–night temperature and light cycle of 19: 15 °C and 15: 9 h, respectively. Daytime illumination was produced by daylight-mimicking light LED panels (approx. 4500 lx at the soil surface level, 4000 K), mounted above each pipe facing downwards. Each mesocosm was weighed every 3 days and water added to maintain constant soil moisture. It should be noted that this experiment was first designed as a 35-day run but progressed to 45 days in the end (Fig. S2). This was because there were 10 nights when earthworms engaged in mating activities. *L. terrestris* are less light sensitive during conjugation and can continue copulating on the soil surface for 1–3 h after sunrise (Darwin 1892). Therefore, these 10 days were removed from the analyses. To ensure equal replications among ALAN treatments, those 10 days were sequentially added to the last day of the experiment. This removal-re-addition process was repeated until no more mating activities occurred. At the end of the experiment, earthworms were retrieved to determine the survival rate by destructively sampling the mesocosms.

#### Experiment 2

This experiment was conducted using the same mesocosm design as for Experiment 1, but was conducted outdoors. The treatments were a 2 × 2 factorial design: earthworm densities (zero and non-zero) and artificial nighttime lighting (0 and 10 lx), each with six replicates arranged in a randomised block design. Two earthworm-free mesocosms (one with and one without artificial light) were placed between the treatments and monitored for stability of ALAN conditions using a data logger (HOBO Pendant MX loggers MX2202, Onset Computer Corp., Bourne, MA).

Earthworms were pre-treated as in Experiment 1, but cultured outdoors under natural light cycles, followed by the same starvation procedure and weight allocation. After three earthworms were added to each mesocosm (average live mass 4.449 g each) and static-cultured for 50 h, each mesocosm was supplied with a mix of 1.50 g *Q. robur* + 1.50 g T*. cordata* leaf litter in a 4 × 4 cm litter bag (3–4 mm mesh). Leaf litter was shredded to 2–4 mm size and moistened two days prior to the experiment. As based on previous observations, *L. terrestris* tend to burrow near soil-pipe borders, the litter bag was placed in the centre of the soil surface to minimise its shading effect. According to the nighttime video recordings, the number of earthworms appeared to decline drastically across mesocosms over time. A further three individuals of similar weight were introduced on the 16th day to each mesocosm formerly inoculated with earthworms.

Two webcams each were assigned to the earthworm-inoculated mesocosms under ALAN treatments of 10 and 0 lx (referred to as lit and unlit treatments thereafter), with the same setup as that in Experiment 1. To cover all replicates in a three-day cycle, each webcam was re-allocated across mesocosms on a daily basis.

This experiment was run in the field, on Penryn Campus, University of Exeter (50°10' N, 5°7' W), for 36 days. There was no artificial lighting in the vicinity that affected the illumination of the mesocosms. Each mesocosm was positioned 0.5 m away from the others to avoid receiving light spillage. Additions of water were administered every 2 to 3 days after weighing the mesocosms to sustain a consistent moisture level. No additional leaf litter was added as food sources. At the end of the experiment, any remaining litter in the litter bags was collected, and earthworms were sampled.

### Analyses

#### Recording of nighttime surface behaviour

For Experiment 1, recordings of earthworm surface behaviour were made after switching off daylight-mimicking lights to induce artificial darkness (22:00–7:00). Throughout Experiment 2, due to changes in day length, the time when the LED lights were on and off triggered by the light-sensitive switch changed from 20:13 to 21:17 h and from 05:24 to 06:35 h, respectively. Therefore, for Experiment 2, the earthworm nighttime behaviour was recorded between 21:30 and 05:00. In addition, six nights were excluded from the analysis where the light levels in the lit treatments dropped below 10 lx due to technical issues (Fig. S3), so the second dataset had 30 nights (120 observations) in total. All webcam materials were analysed at 1-min intervals and reviewed by one person (J.C.) using a standard media player.

In both experiments, the surface emergence of an earthworm to any degree, from exposure of the anterior body segments to complete emergence from the burrow, was counted as overall surface behaviour (‘Overall’ hereafter). As a further measure of behavioural differences, we subdivided earthworm surface behaviour into three categories, when (a) only the anterior tip of the body rapidly rose and fell (‘Heads-only’ hereafter), (b) its body bent parallel to the soil surface and started crawling, yet with incomplete emergence from the burrow (‘Half-emerged’ hereafter), and (c) fully emerged. We did this for the following two reasons. First, the light-sensitive response of *L. terrestris* may vary depending on their body exposure under ALAN. This is conceivable given there is an imbalanced distribution of photoreceptors (both in epidermis and nerve enlargements), with the most abundant in the prostomium area (Hess 1925). Second, *L. terrestris* showed clear behavioural differences amongst these three different levels of exposure; for example, foraging activities were limited to the time between the anterior tip exposure and full emergence, whereas over-surface exploration (Butt et al. 2003) was limited to and was the only event during which they were fully emerged. Therefore, the three behaviours can be seen as measures, respectively, of the extent to which (a) the exploratory behaviour for risk was interrupted, (b) the foraging activity was affected and (c) the over-surface exploration was disrupted.

#### Measurement of leaf litter removal

Leaf litter removal (*R*) from the litter bags was measured at the end of Experiment 2. All litter was dried in a forced-air drying oven for 24 h at 105 °C and weighed. All dried litter mass was multiplied by a conversion factor of 1.12 (derived by oven-drying a set of air-dried litter samples) to attain the remaining litter mass. Given the experiment was run in a non-invertebrate-free environment in the field, we corrected the remaining mass of leaf litter in the earthworm-inoculated mesocosms with that in the corresponding control to account for the leaf litter consumption only induced by earthworm behaviour. Correction was made following the Reimann formula (Szlavecz 1993):

$$R=\frac{(M-m)S}{M}$$

where *S* is the initial mass of the leaf litter, and *m* and *M* are the remaining mass of the leaf litter in the lit (or unlit) treatment and the corresponding control, respectively.

#### Measurement of soil respiration rate

Seven measurements of soil respiration rate were carried out after Experiment 2 had started (namely, consecutively on the first 2 days and every other week after Day 1). Here, the reported soil respiration refers to dark respiration specifically. Measurements were taken during the day to preclude the confounding effects of artificial lighting. To eliminate potential photorespiration in the daytime, dark treatment was achieved by covering the mesocosms with two-ply black sack bags under which complete darkness was induced to the human eye. To obtain dark respiration rate, an in situ measure of soil CO_2_ efflux was performed using a portable infrared gas analyser (Q-Box SR1LP Soil Respiration Package, Qubit Systems, Canada) equipped with G180 Soil Chamber. During the measurements, the soil chamber was placed on top of the soil and sampled the CO_2_ concentration twice per second.

#### Statistical analyses

All statistical analyses were conducted in R (v. 4.2.2; R Core Team 2022). Each model was carefully assessed to ensure the key assumptions were met; for example, behaviour data were square-root transformed if not specifically noted.

Nighttime surface behaviour data from Experiment 1 were analysed with a linear mixed effect model using the *lme4* package (Bates et al. 2015). Each behaviour type (i.e. overall, heads-only and half-emerged) was analysed separately, with seven ALAN treatments as a fixed effect, and pipe ID and time included as random effects. The effect size and a 95% confidence interval for each behaviour type per ALAN level were extracted using the *confint* function (*stats* package; R Core Team 2022). Due to insufficient observations (3 out of 140), the full-emerged surface behaviour was not considered for further analysis.

Nighttime surface behaviour data from Experiment 2 were found to be zero-inflated using the *DHARMa* package (Hartig 2022), after checking for potential dispersion issues. Thus, they were analysed with a generalised linear mixed model that included a zero-inflated component using the *glmmTMB* package (Brooks et al. 2017). Each behaviour type was modelled separately, with ALAN treatments as a fixed effect, and pipe ID and time included as random effects. Error structure (Poisson or negative binomial) for each model was determined following data dispersion evaluation and model performance comparison (indicated by AIC). The data pertaining to the fully emerged surface behaviour were limited to eight mesocosms with not enough observations (14 out of 120), resulting in severe underdispersion during model fitting, and therefore were removed from subsequent analysis.

Worm-induced leaf litter removal was analysed with a generalised linear model (Gaussian error structure). Given earthworm survival was highly correlated, the model with ‘Treatments’ as an explanatory variable and ‘Treatment: Survival’ as an interaction term was considered.

Soil respiration was found to differ between treatments when *L. terrestris* were absent (Fig. S4). Therefore, we used worm-induced soil respiration through estimation by subtracting the CO_2_ emitted by the corresponding control from that emitted by the earthworm-inoculated mesocosms under lit or unlit treatments. Significance of the difference in worm-induced changes in soil respiration between treatments was tested using ANOVA or Mann–Whitney *U* test. Normality of data was checked (Shapiro–Wilkes test), followed by homogeneity of variance diagnosis (Levene’s test or Bartlett’s test). We found earthworm survival had limited impacts on soil respiration (Table S1) and therefore was not into the analyses.

## Results

### Experiment 1

#### Earthworm survival

All earthworms remained alive throughout the course of the experiment in all except for two mesocosms, where one individual each was dead.

#### Behavioural responses to seven levels of ALAN

**Fig. 1:**
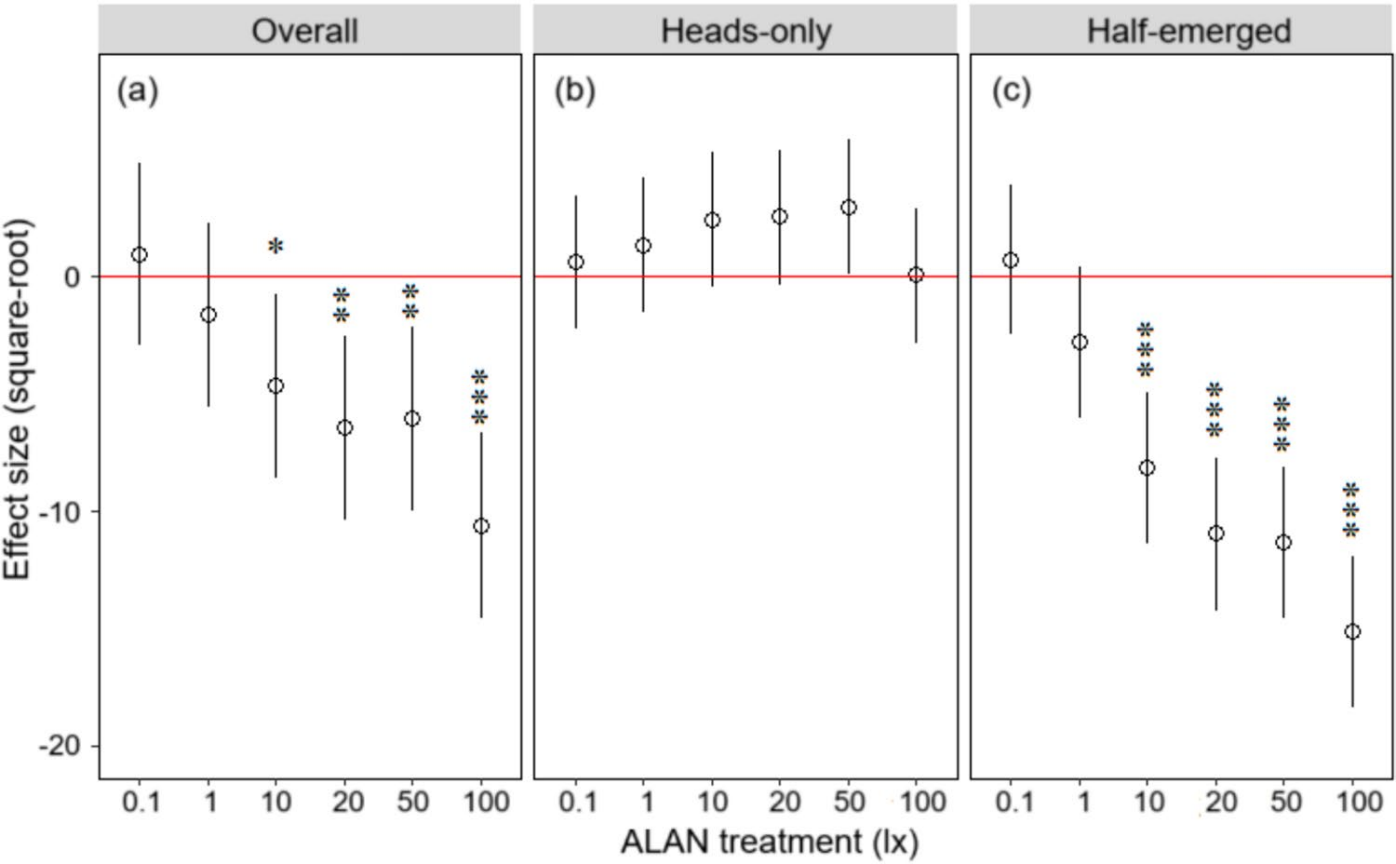
Effect size of ALAN treatment (square-root transformed) relative to the dark control on *L. terrestris* nighttime surface behaviour: **a** overall, **b** heads-only, and **c** half-emerged in Experiment 1. Hollow dots indicate parameter estimates, associated lines indicate 95% confidence intervals, and stars indicate effects significantly differing from zero (*P < 0.05, **P < 0.01, ***P < 0.001)

Compared to the dark control, ‘Overall’ and ‘Half-emerged’ nighttime surface behaviour dropped considerably at ALAN levels of at least 10 lx (Fig. 1a and c). As light intensity increased, the negative ALAN effect sizes (after square-root transformation) enhanced approximately linearly. However, there was no significant difference detected for ‘Heads-only’ behaviour regardless of light intensity (Fig. 1b).

### Experiment 2

#### Earthworm survival

During Experiment 2, 49 out of 72 earthworms survived (survival rate: 68.1%). Earthworm survival was slightly, but not significantly, lower in the lit treatments than the unlit (61.1% relative to 75.0%, *P* > 0.05).

#### Behavioural responses to ALAN level of 10 lx in the field

**Fig. 2:**
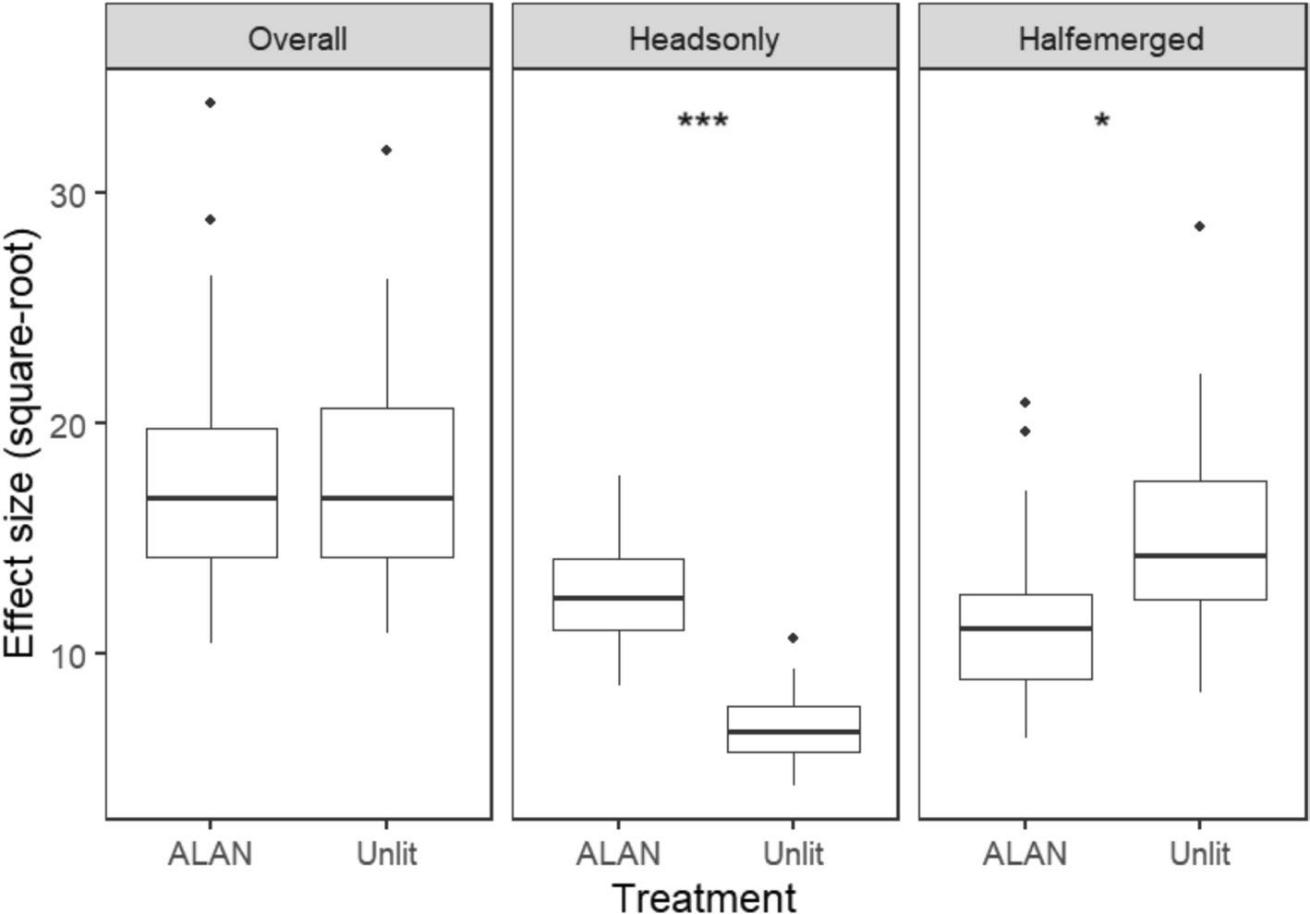
Effect size of treatments (square-root transformed) on *L. terrestris* nighttime surface behaviour (overall, heads-only, and half-emerged) in Experiment 2. Stars indicate significant differences between treatments (*P < 0.05, ***P < 0.001). Note: these effect sizes were extracted from the conditional part of zero-inflated models. The effect size from the zero-inflation part that describes the probability of observing an extra zero that is not generated by the conditional model was not shown, because there was no significant difference between treatments

Under the ALAN level of 10 lx in the field, ‘Heads-only’ and ‘Half-emerged’ behaviour exhibited contrasting responses (Fig. 2). Specifically, relative to the unlit control, there was significantly more ‘Heads-only’ behaviour as indicated by the positive estimate from the conditional model (*P* < 0.001), whereas there was considerably less ‘Half-emerged’ behaviour as suggested by the negative conditional model estimate (*P* < 0.05). There was no detectable difference in the ‘Overall’ behaviour (*P* > 0.05), likely because of the opposing changes in ‘Half-emerged’ and ‘Heads-only’ behavioural responses.

#### Leaf litter removal

In earthworm-inoculated mesocosms, the corrected leaf litter removal in the lit and unlit treatment was 0.66 ± 0.26 and 0.46 ± 0.20 g, respectively. Despite fewer *L. terrestris* surviving at the end of Experiment 2, the leaf litter removed in the lit treatment was, on average, 42.4% higher than the unlit, suggesting that *L. terrestris* under the ALAN level of 10 lx contributed to the majority of the leaf litter loss. When further accounting for earthworm survival, the higher earthworm survival in the lit treatment can lead to a significant, albeit marginal, increase in leaf litter removal (*P* = 0.049), whereas no such effect was observed in the unlit treatment (*P* = 0.271).

#### Soil respiration

**Fig. 3:**
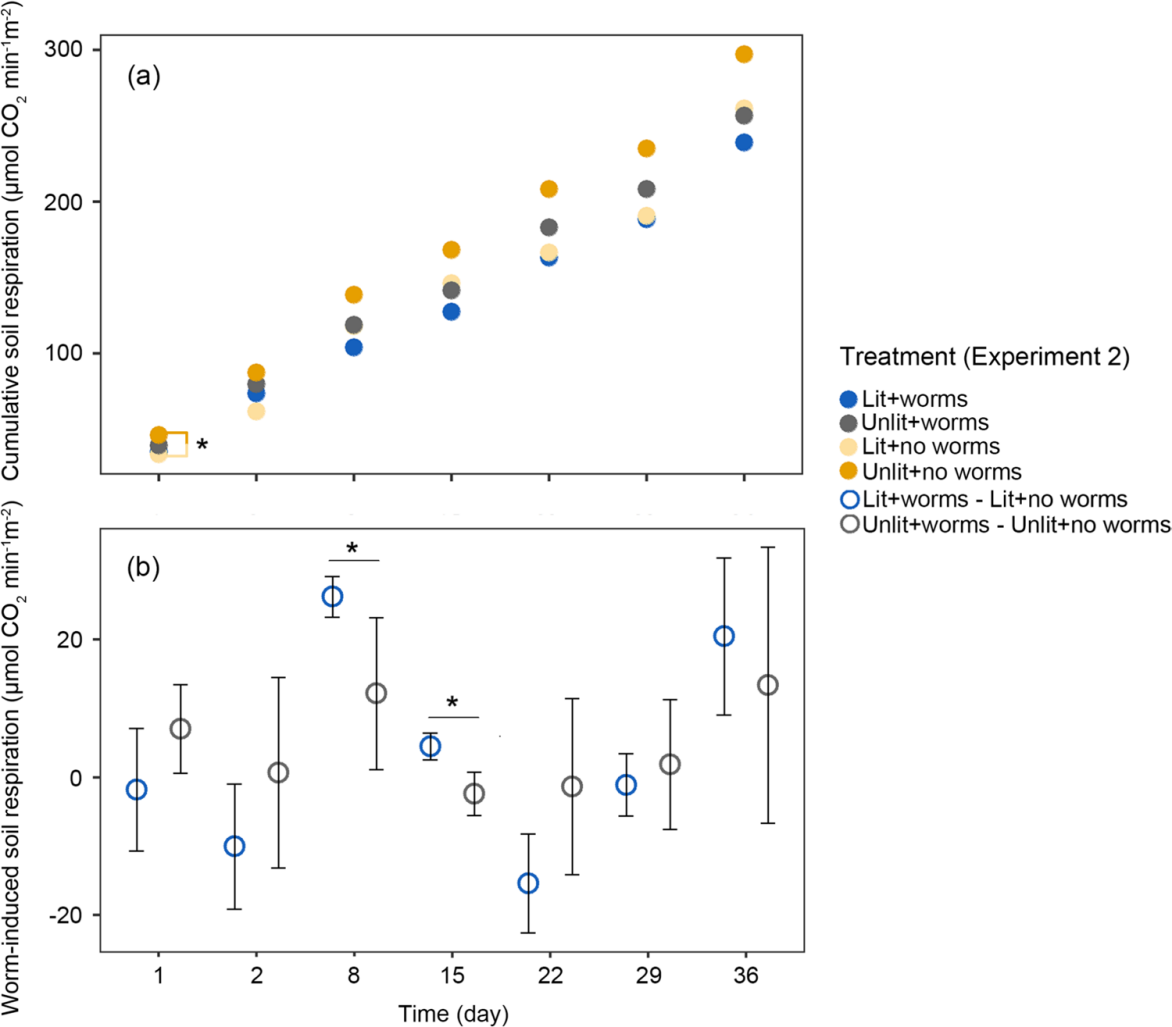
Changes in **a** cumulative soil respiration and **b** worm-induced soil respiration over time in Experiment 2. Asterisks denote significant differences between treatments at *P* < 0.05. Error bars indicate standard error

There were only a few statistically significant differences detected in the worm-induced soil respiration across seven measurements (Fig. 3). Compared to the corresponding control, CO_2_ levels were reduced by *L. terrestris* surface behaviour over 36 days to 238.8 ± 33.1 and 256.6 ± 46.2 μmol CO_2_ min^−1^ m^−2^ in the lit and unlit treatments, respectively (Fig. 3a). Worm-induced soil respiration for each treatment fluctuated with time, with a greater magnitude of variations in soil respiration in the lit treatment than in the unlit (Fig. 3b). In the near mid-point of the experiment (Day 8 and 15), worm-induced soil respiration in the lit treatment was significantly higher compared with that in the unlit (*P* < 0.05, Fig. 3b).

## Discussion

To our knowledge, this study is the first experimentally to provide direct evidence (via continuous, real-time recording) of earthworm behavioural responses to ALAN and to explore a connection between these behavioural changes and C cycling. We found that an ALAN level of 10 lx was the minimum (or near-minimum) required significantly to disrupt *L. terrestris* nighttime behaviour on the soil surface. However, despite this behavioural change, we did not observe corresponding alterations in leaf litter removal or soil respiration.

Behavioural monitoring of earthworms under ALAN in both controlled-temperature (Experiment 1) and field conditions (Experiment 2) revealed that reduced nighttime surface behaviour in *L. terrestris* (esp. foraging time) occurred at levels of 10 lx and above. Such negative ALAN effect sizes (square-root transformed) grew as light intensity increased (Fig. 1), which is similar to previous studies examining earthworm escape responses to intensely bright light (e.g. *L. terrestris*—Hess 1924; *Eisenia fetida*—Lin et al. 2018; Prosser 1934). Although 10 lx was the minimum limit at which an effect on foraging on the soil surface was detected (Figs. 1 and 2), it appears still above the lowest threshold required to induce a reduction in *L. terrestris* foraging activity based on the ALAN-behaviour relationship in Experiment 1. Recently, a study by Mittmannsgruber et al. (2024) reported indirect evidence of reduced earthworm surface activity under exposure to ALAN of 5 lx for 45 days, finding that the number of surface casts produced and toothpicks (inserted 2–3 mm vertically into the soil) moved by *L. terrestris* were reduced by 37% and 76%, respectively (relative to the dark treatment). Previously, it has been suggested that short-term exposure to artificial light (within 24 h), levels of which were close to 1 lx or even lower, can trigger an escape response in *L. terrestris* (Hess 1924). As such, reduction in earthworm surface activity may not be confined to the more brightly lit areas near streetlights but could occur further away (e.g. areas outside of 20 m from a typical street lamp; Bennie et al. 2016). With the ongoing expansion of ALAN (estimated rate: 2.2% per year; Kyba et al. 2017), this reduction may become more prevalent across a broader area. Previously, using spatial datasets that provided the actual distribution of streetlights across parts of the UK, one study estimated that *c.* 1.1% of the 6244 km^2^ land area (e.g. green spaces within towns and cities, grasslands and woodlands) had already been directly exposed to ALAN of > 1 lx (Boyes et al. 2021).

Whilst the two experiments in this work exhibited similarities in the negative responses of *L. terrestris* nighttime surface foraging to 10 lx, they presented differences in the response of the exploratory behaviour for risk (i.e. ‘Heads-only’ behaviour). Specifically, relative to the control, there was no observable difference in the risk exploratory behaviour regardless of light intensity in *L. terrestris* in Experiment 1, whereas there were significantly more attempts for risk assessment before surfacing in Experiment 2. There might be two main causes. First, the behavioural differences could be driven by between-experiment dissimilarity in pressures induced by ALAN and environmental conditions. The interactions between ALAN and temperature (demonstrated to act synergistically on predation and parasitism—Kehoe et al. 2020; Miller et al. 2017—and life-history traits—Nguyen et al. 2020), for example, may be one of the factors that contributed to such differences. Unlike the consistent temperature regimes applied throughout Experiment 1 (day: night = 19: 15℃), temperature conditions in Experiment 2 varied in magnitude (well below 15℃ throughout all nights) and amplitude (T_avg_ = 9.4℃ [T_min_–T_max_ = 4.8–13.1℃], Supplementary Fig. S5 and S6). In a previous study under conditions of a slightly wider temperature range (T_avg_ = 8.8℃) than in our case, *L. terrestris* emergence was positively associated with temperature (Nuutinen et al. 2014), whilst another study with presumably higher average night temperatures found the opposite (Macdonald 1983). In addition, prior observations suggest that the optimal temperature was 22℃ for *L. terrestris* (juvenile) food intake and 15℃ for growth and reproduction (Butt 1991; Daniel et al. 1996). As such, driven by the life-history trade-off, *L. terrestris* in Experiment 2 may have attempted more surface risk assessment before foraging, balancing the need to feed against pressures from both low air temperatures and ALAN. Second, the behavioural differences could also be driven by inconsistent temporal effects of ALAN. Fixed-moving-window analyses suggested that significantly more risk exploratory behaviour under ALAN was only limited to the time shortly after *L. terrestris* were inoculated into the mesocosms (Experiment 2, Supplementary Fig. S7 and S8). Unlike the consistent ALAN level throughout Experiment 2, randomised nighttime light levels across days in Experiment 1 may thus have masked the temporal effects of ALAN on early risk exploratory behaviour regardless of light intensity.

Taken together, our findings from both experiments underscore that ALAN, at the level of 10 lx (or potentially lower), is sufficient to cause disruptions to *L. terrestris* nighttime surface behaviour. However, attention should be paid here to potential light adaptation. Even though there are limited records of earthworm adaptation to light, a study by Hess (1924) showed that prolonged exposure to artificial bright light can lead to desensitisation of photoreceptors in *L. terrestris*, which reduces their immediate light aversion. Albeit likely confounded by earthworm mortality, fixed-moving-window analyses in this study indicated potential light adaptation in *L. terrestris* (Supplementary Fig. S7 and S8). As such, further exploration of the effects of low-level artificial nighttime lighting for longer periods in the field would be informative.

It is well known that earthworm activity is a crucial driver of organic matter breakdown and carbon fluxes in ecosystems. Whilst we did not detect discernible changes in either litter removal or soil respiration under 10 lx ALAN (Fig. 3, Table S1), controlled experiments reported that *L. terrestris* decreased litter removal by 51% and soil aggregate production by 37% under 5 lx nighttime lighting (Mittmannsgruber et al. 2024; Kavassilas et al. 2024) and that ALAN around 10 lx level can affect microbial abundance (Hölker et al. 2015). This raises the possibility of complex interactions between earthworm behaviour and soil microbes on C cycling under ALAN. In fact, earthworms can stabilise C by forming soil aggregates through their feeding and burrowing activity (Bossuyt et al. 2005), or conversely increase C mineralisation by stimulating soil microbial activity, which contributes 17–32.2% of soil organic matter mineralisation (Tardy et al. 2015). Such opposing mechanisms make the effect of earthworms on carbon cycling closely tied to that of soil micro-organisms and appear to be time-sensitive (Zhang et al. 2013); for instance, an endogeic earthworm species (*Octolasion tyrtaeum*) reduced CO_2_ production by 15–39% only in studies exceeding 100 days (Scheu 1997; Marhan and Scheu 2005b; Scheu and Wolters 1991). But still, the intricate interplay between earthworms and soil microbes under ALAN and how this may affect C cycling remain largely unknown, and further research is needed to establish underlying dose–response or threshold relationships over different timescales.

## Supplementary Information

Below is the link to the electronic supplementary material.

Supplementary file1 (DOCX 532 KB)

## Data Availability

The datasets used and/or analysed during the current study are available from the corresponding author on reasonable request.

## Code availability

We have not archived our analysis code.
