# Supplementary material for: Altered surface behaviour in earthworms (Lumbricus terrestris) under artificial light at night
Source: Oecologia. 2025 Jun 27;207(7):114. doi: 10.1007/s00442-025-05750-z (PMC12205020; doi:10.1007/s00442-025-05750-z)
Supplement: Supplementary file 1 — Supplementary file1 (DOCX 532 KB) [file 442_2025_5750_MOESM1_ESM.docx]

**Altered surface behaviour in earthworms (*Lumbricus terrestris*) under artificial light at night**

**Jiaqing Cai^1*^, Jonathan Bennie^2^ and Kevin J. Gaston^1^**

^1^ Environment and Sustainability Institute, University of Exeter, Penryn, Cornwall TR10 9FE, UK

^2^ Centre for Geography and Environmental Science, University of Exeter, Penryn, Cornwall TR10 9FE, UK

^*^E-mail: jc1429@exeter.ac.uk


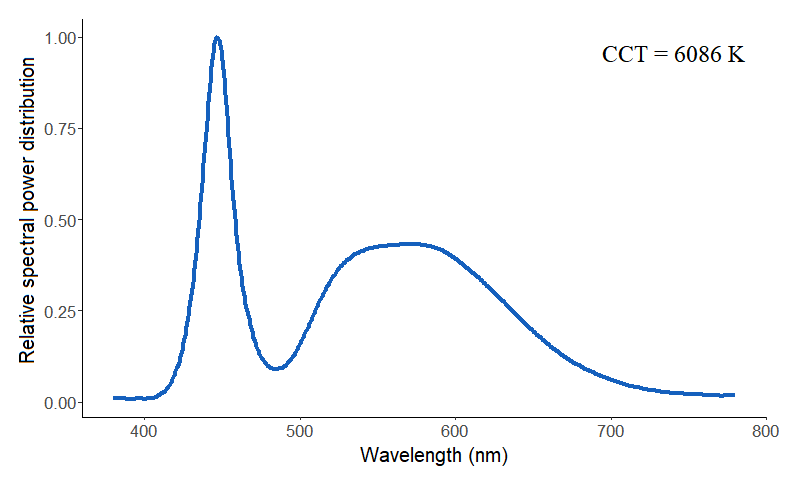


**Fig. S1** Relative spectral power distribution of the white LED lights used in both experiments for this study. The spectrum was measured using a spectrometer (UPRtek MK350N PLUS spectrometer, United Power Research Technology Corporation).


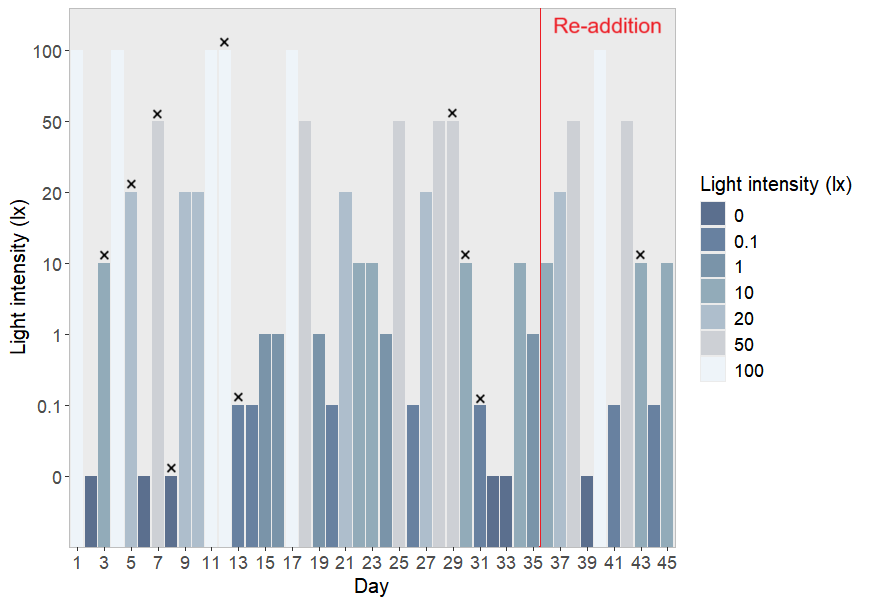


**Fig. S2** Randomized repeated measures design for the seven ALAN treatments in Experiment 1. × denotes days when there were mating activities of *L. terrestris* on the soil surface.


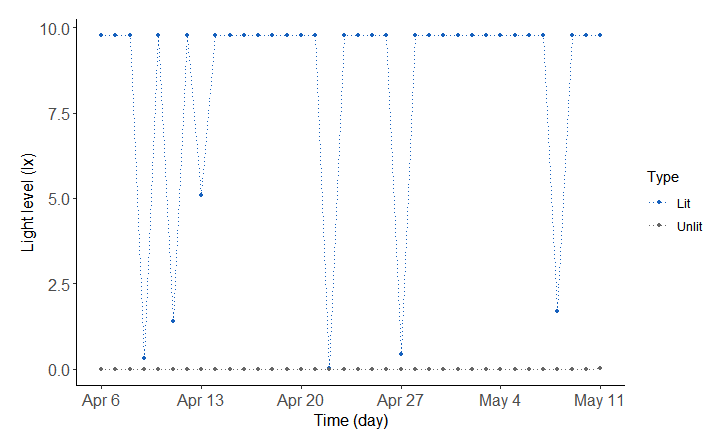


**Fig. S3** Average artificial nighttime light levels (09:30 pm - 05:00 am) in the lit and unlit treatments throughout Experiment 2


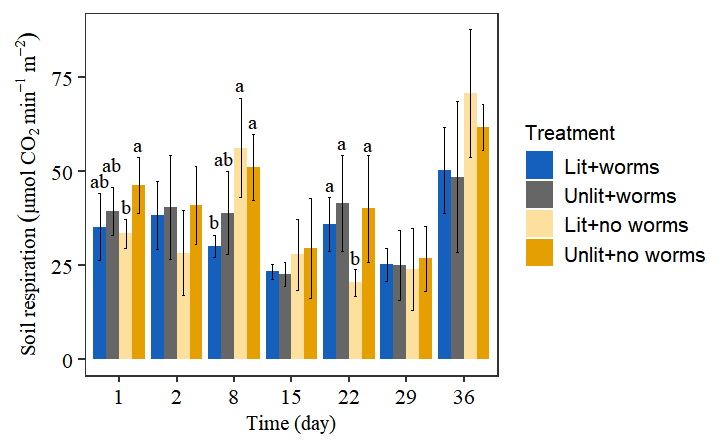


**Fig. S4** Soil respiration in the earthworm-inoculated and -free mesocosms under lit and unlit treatment over time (Experiment 2). Different letters denote detected differences by ANOVA or Kruskal-Wallis test at *p* < 0.05 significance level.


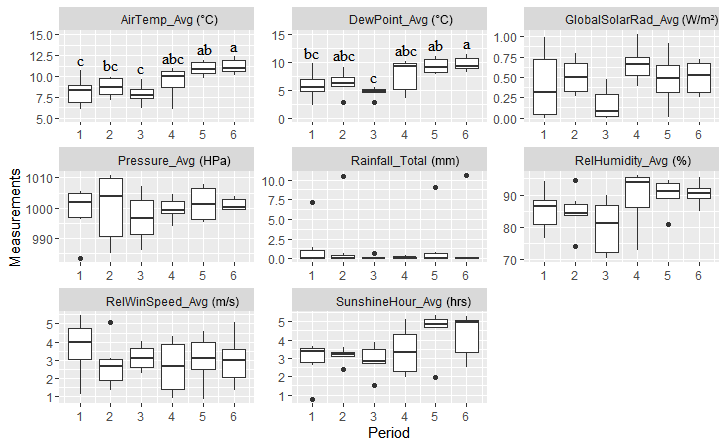


**Fig. S5** Eight meteorological measurements with a fixed moving window (6-day period, non-overlapping) over 36 days (Experiment 2). All measurements were taken during 09:30 pm – 05:00 am (the next day) from the nearest weather station (50°10'16"N 5°07'40"W), except for the total precipitation which was derived from the rainfall station (50°9'37"N, 5°6'52"W). Tests for differences between periods were conducted using ANOVA followed by LSD multiple comparison analysis. Where there was a significant difference (*p* < 0.05), letters were shown above each boxplot.

**
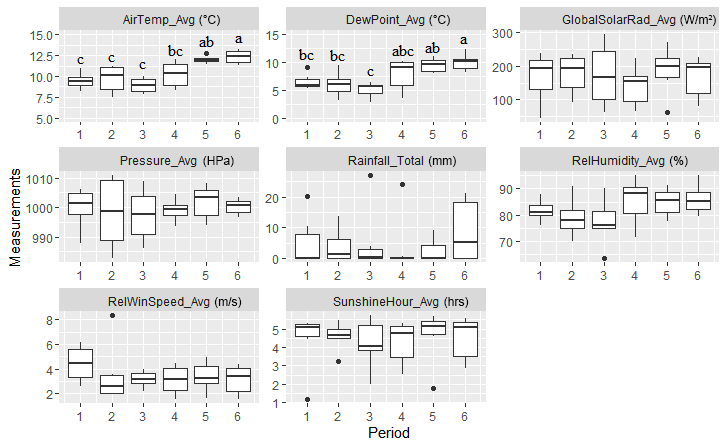
**

**Fig. S6** Eight meteorological measurements with a fixed moving window (6-day period, non-overlapping) over 36 days (Experiment 2). All measurements were taken during 05:00 am – 05:00 am (the next day) from the nearest weather station (50°10'16"N 5°07'40"W), except for the total precipitation which was derived from the rainfall station (50°9'37"N, 5°6'52"W). Tests for differences between periods were conducted using ANOVA followed by LSD multiple comparison analysis. Where there was a significant difference (*p* < 0.05), letters were shown above each boxplot.


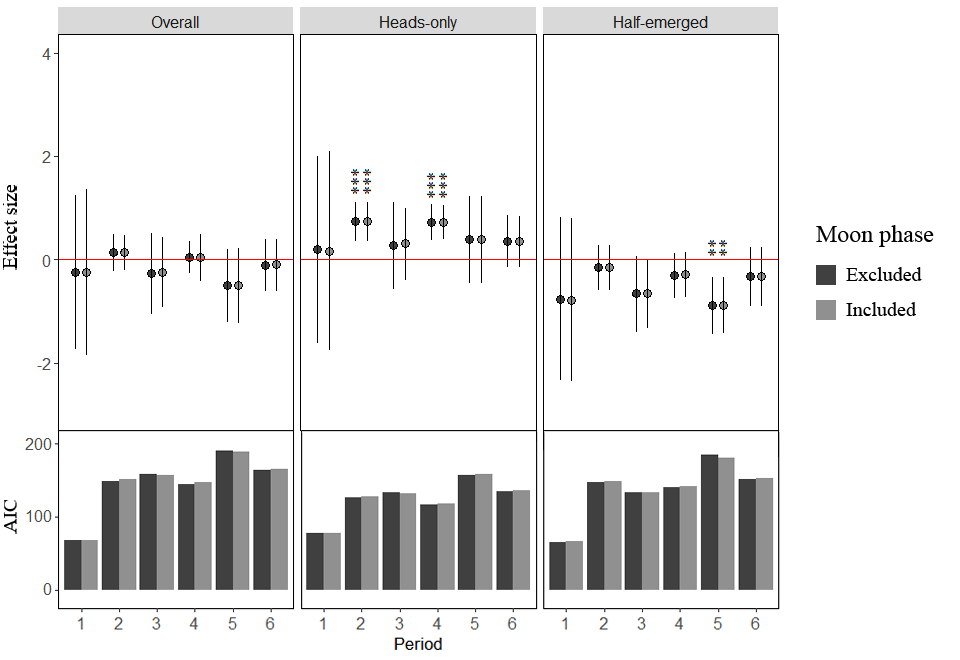


**Fig. S7** Changes in effect size and model’s goodness of fit (indicated by AIC) of ALAN level of 10 lx relative to the unlit control on *L. terrestris* nighttime surface behaviour (a) Overall, (b) Heads-only, and (c) Half-emerged using a fixed moving window (6-day period, non-overlapping) over 36 days in Experiment 2 (09:30 pm – 05:00 am the next day). Activity data were transformed where necessary. Activity data for each period were fitted with a generalised linear mixed model (error structure: Poisson/negative binomial/COM-Poisson) using Treatment as a fixed effect, and Pipe ID and Night as random effects. Because temperature and precipitation were associated with earthworm activity (Nuutinen et al., 2014), they entered as covariates to account for the difference in night conditions (Dewpoints varied greatly over 6 periods, but they had a strong correlation (r = 0.9) with temperature and were therefore excluded). Moon phase was included or excluded as a covariate to compare model performance, as it has been suggested to affect *L. terrestris* activity (Michiels et al., 2001). Hollow dots indicate parameter estimates, associated lines indicate 95% confidence intervals, and stars indicate effects significantly differing from zero (***p* < 0.01, ****p* < 0.001).


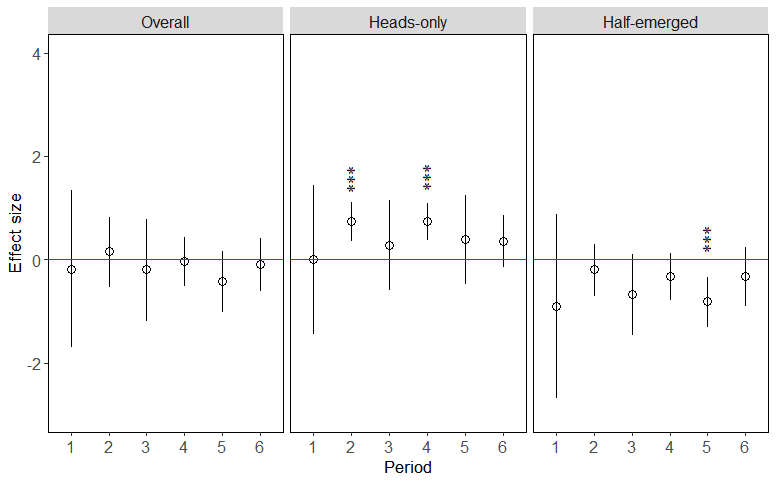


**Fig. S8** Changes in effect size of ALAN level of 10 lx relative to the unlit control on *L. terrestris* nighttime surface behaviour (a) Overall, (b) Heads-only, and (c) Half-emerged using a fixed moving window (6-day period, non-overlapping) over 36 days in Experiment 2 (05:00 am – 05:00 am the next day). Activity data were transformed where necessary. Activity data for each period were fitted with a generalised linear mixed model (error structure: Poisson/negative binomial/generalized Poisson/COM-Poisson) using Treatment as a fixed effect, and Pipe ID and Night as random effects. Because temperature and precipitation were associated with earthworm activity (Nuutinen et al., 2014), they entered as covariates to account for the difference in night conditions (Dewpoints varied greatly over 6 periods, but they had a strong correlation (r = 0.87) with temperature and were therefore excluded). Hollow dots indicate parameter estimates, associated lines indicate 95% confidence intervals, and stars indicate effects significantly differing from zero (****p* < 0.001).

**Table S1.** Effects of ALAN treatment and earthworm survival on cumulative soil respiration

|  | DF | Sum Sq | Mean Sq | F value | Pr(>F) |
| --- | --- | --- | --- | --- | --- |
| Day 29 |  |  |  |  |  |
| Treatment | 1 | 1157.8 | 1157.84 | 1.2279 | 0.3000 |
| Treatment:Survival | 2 | 3091.7 | 1545.84 | 1.6394 | 0.2531 |
| Day 36 |  |  |  |  |  |
| Treatment | 1 | 957.7 | 957.67 | 0.5562 | 0.4771 |
| Treatment:Survival | 2 | 5591.1 | 2795.56 | 1.6238 | 0.2559 |

**References**

Michiels NK, Hohner A, Vorndran IC (2001) Precopulatory mate assessment in relation to body size in the earthworm *Lumbricus terrestris*: avoidance of dangerous liaisons? *Behav. Ecol.* **12**, 612–618. (doi: 10.1093/beheco/12.5.612)

Nuutinen V, Butt KR, Jauhiainen L, Shipitalo MJ, Sirén T (2014) Dew-worms in white nights: High-latitude light constrains earthworm (*Lumbricus terrestris*) behaviour at the soil surface. *Soil Biol. Biochem.* **72**, 66–74. (doi: 10.1016/j.soilbio.2014.01.023)
